# Three-dimensional ultrasound manifestations of adenomyosis

**Published:** 2013-10

**Authors:** Firoozeh Ahmadi, Hadieh Haghighi

**Affiliations:** *Department of Reproductive Imaging at Reproductive Biomedicine Research Center, Royan Institute for Reproductive Biomedicine, ACECR, Tehran, Iran.*

Adenomyosis is an idiopathic and benign disease in which ectopic endometrial glands embedded deeply in myometrial smooth muscle with the compensatory hypertrophy of the myometrium surrounding the ectopic endometrium (1, 2). There is a correlation between depth of the infiltration and initial too high stage of adenomyosis. Lesions may characterize as a diffuse or focal lesions. The highest prevalence occurs in parous women 30-50 years old and in half of the cases remains asymptomatic (2, 3). By now, there are no definitive detection methods for adenomyosis and the condition still remains a diagnostic challenge. Transvaginal ultrasound (TVS) is the primary imaging modality for preoperative diagnosis of diffuse adenomyosis. 

There is a broad spectrum of ultrasound features in adenomyosis. Typical adenomyosis on two-dimensional ultrasound (2D-TVS) is characterized by the presence of focal or diffuse myometrial heterogeneity (4, 5). Diffuse adenomyosis represents as asymmetric myometrial thickening and/or enlarged and globular uterus. Other findings in adenomyosis include echogenic nodules or linear striations extending from the endometrium into the myometrium results from presence of ectopic endometrial glands and stroma and also dilated cystic glands result in small myometrial cysts(1). 

**Figure 1 a, b.**
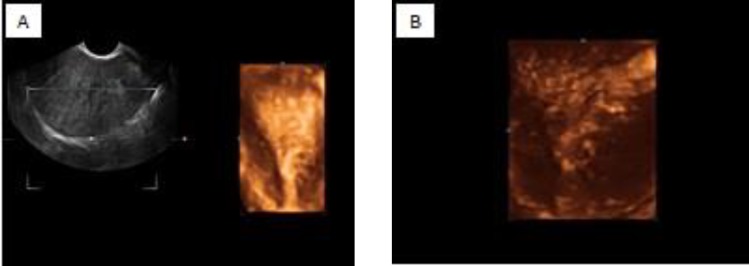
Three-dimensional ultrasound showing the complete infiltration of the junctional zone

In a recent review Reinhold and colleagues reported that TVS had a sensitivity of 80-86%, specificity of 50-96%, and overall accuracy of 68-86%for diagnosing diffuse adenomyosis (1). However, TVS can yield equivocal result in the case of focal adenomyosis and if there are co-existent fibroids (6). Furthermore, the 2D-TVS findings are more likely appears in advanced stage of disease. With the advent of real time three-dimensional ultrasound (3D-TVS) the diagnostic potential of ultrasound examination of adenomyosis has improved significantly. The coronal section of the uterus and post processing arrangement provided by 3D-TVS has led to substantial improvement in the diagnosis of adenomyosis on early stage (7, 8).

Recent studies showed that the irregular endometrial-myometrial junction (junctional zone, JZ) with increased thickness depicted on coronal view of 3D-TVS as a sonographic criteria has a high diagnostic accuracy particularly for diagnosis of initial adenomyosis based on the reasonable hypothesis that adenomyosis is caused by infiltration of endometrial tissue across the junctional zone and into myometrium(7, 9). Ahmed *et al* found the positive predictive value (PPV) and accuracy of 3D-TVS in the diagnosis of adenomyois to be 95% and 80%, respectively, based on detection of irregular junctional zone on coronal plane. This picture of month aims to illustrate a typical feature of adenomyosis on three-dimensional ultrasound (Figure 1 a, b). 3D-TVS provide the most comprehensive and detailed view of the uterus and improves the evaluation of adenomyosis in the office as a first line diagnostic tool.

## References

[1] Reinhold C, Tafazoli F, Mehio A, Wang L, Atri M, Siegelman ES (1999). Uterine adenomyosis: endovaginal US and MR imaging features with histopathologic correlation. Radiographics.

[2] Jeng CJ, Huang SH, Shen J, Chou CS, Tzeng CR (2007). Laparoscopy-guided myometrial biopsy in the definite diagnosis of diffuse adenomyosis. Hum Reprod.

[3] Azziz R (1989). Adenomyosis: current perspectives. Obstet Gynecol Clin North Am.

[4] Reinhold C, Atri M, Mehio A, Zakarian R, Aldis AE, Bret PM (1995). Diffuse uterine adenomyosis: Morphologic criteria and diagnostic accuracy of endovaginal sonography. Radiology.

[5] Fedele L, Bianchi S, Dorta M, Arcaini L, Zanotti F, Carinelli S (1992). Transvaginal ultrasonography in the diagnosis of diffuse adenomyosis. Fertil Steril.

[6] Perrot N, Frey I, Mergui JL, Bazot M, Uzan M, Uzan S (2001). Picture of the month. Adenomyosis: power Doppler findings. Ultrasound Obstet Gynecol.

[7] Exacoustos C, Brienza L, Di Giovanni A, Szabolcs B, Romanini ME, Zupi E (2011). Adenomyosis: three-dimensional sonographic findings of the junctional zone and correlation with histology. Ultrasound Obstet Gynecol.

[8] Naftalin J, Jurkovic D (2009). The endometrial-myometrial junction: a fresh look at a busy crossing. Ultrasound Obstet Gynecol.

[9] Kepkep K, Tuncay YA, Göynümer G, Tutal E (2007). Transvaginal sonography in the diagnosis of adenomyosis: which findings are most accurate?. Ultrasound Obstet Gynecol.

